# DNA Methylation in Skeletal Muscle Stem Cell Specification, Proliferation, and Differentiation

**DOI:** 10.1155/2016/5725927

**Published:** 2016-01-05

**Authors:** Rhianna C. Laker, James G. Ryall

**Affiliations:** ^1^Department of Medicine, Center for Skeletal Muscle Research at the Robert M. Berne Cardiovascular Research Center, University of Virginia, Charlottesville, VA 22908, USA; ^2^Stem Cell Metabolism & Regenerative Medicine Group, Basic & Clinical Myology Laboratory, The University of Melbourne, Melbourne, VIC 3010, Australia

## Abstract

An unresolved and critically important question in skeletal muscle biology is how muscle stem cells initiate and regulate the genetic program during muscle development. Epigenetic dynamics are essential for cellular development and organogenesis in early life and it is becoming increasingly clear that epigenetic remodeling may also be responsible for the cellular adaptations that occur in later life. DNA methylation of cytosine bases within CpG dinucleotide pairs is an important epigenetic modification that reduces gene expression when located within a promoter or enhancer region. Recent advances in the field suggest that epigenetic regulation is essential for skeletal muscle stem cell identity and subsequent cell development. This review summarizes what is currently known about how skeletal muscle stem cells regulate the myogenic program through DNA methylation, discusses a novel role for metabolism in this process, and addresses DNA methylation dynamics in adult skeletal muscle in response to physical activity.

## 1. Introduction

The term “epigenetics” literally means “above genetics” and is defined by the NIH Roadmap Epigenomics project as “both heritable changes in gene activity and expression (in the progeny of cells or of individuals) and also stable, long-term alterations in the transcriptional potential of a cell that are not necessarily heritable.” Epigenetics underlies the ability of embryonic stem cells (with an identical DNA code) to commit to the three germ layers (mesoderm, endoderm, and ectoderm) during the early stages of development and eventually commit to specific cell fates to generate all the different cell types in an organism, including skeletal muscle. These biological trait variations are not a result of changes in the DNA code, but rather structural modifications to the DNA and/or histones, or posttranscriptional gene silencing via small RNAs (including miRNA, siRNA, and piRNA) [1].

Considering the interest surrounding epigenetics and in particular DNA methylation, in the regulation of stem cell identity, this review aims to discuss some of the recent findings regarding methylation, with a particular focus on skeletal muscle stem cells (MuSCs, also referred to as satellite cells). While not discussed in this review, it is worth mentioning that, in addition to direct DNA modifications, structural epigenetic control is conferred at the level of histones. The core histone proteins H2A, H2B, H3, and H4 all contain long N-terminal tails which are highly susceptible to posttranslational modifications including methylation (me), acetylation (ac), phosphorylation (p), SUMOylation (sumo), ubiquitination (ub), ADP-ribosylation (ADP), and citrullination (cit) (reviewed in [2]). Each of these modifications influences the structure of the chromatin and directly regulates transcription. The complexity of many of these histone modifications has recently been documented in a series of publications arising from the Roadmap Epigenomics project (selected publications [3–5]).

## 2. DNA Methylation

Before discussing the role of DNA methylation in MuSC biology, it is essential to first define the process of methylation. Methylation of DNA is a well-described phenomenon and primarily occurs on the 5′ position of cytosine bases within CpG dinucleotide pairs and leads to the formation of 5-methylcytosine (5mC) and a context specific effect on transcription. DNA methylation within the promoter region of genes is typically linked to transcriptional repression due to recruitment of methyl CpG binding domain (MBD) proteins, which block transcription factor and RNA polymerase access [6]. In contrast, intragenic DNA methylation has been observed to have variable effects on gene transcription and can regulate the process of alternative splicing [7–9]. Finally, like promoter methylation, intergenic DNA methylation has been linked to gene repression likely as a result of inhibiting the actions of long range gene enhancers [10, 11]. Although research to date has focused on the role of promoter region methylation, the emergence of whole genome sequencing techniques has highlighted the potential for alterations to intragenic and intergenic methylated regions in response to environmental stimuli. Their involvement in the regulation of gene expression programs will greatly enhance our understanding of tissue specific transcriptional programs.

**Figure 1:**
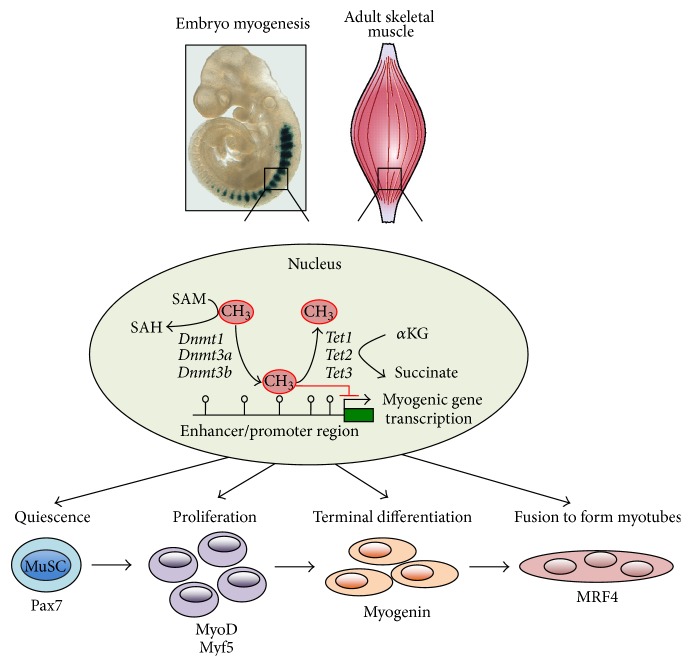
Transient DNA methylation and demethylation via specific* Dnmt* and* Tet* isoforms, respectively, regulate the expression of myogenic genes during embryonic MuSC specification, proliferation, and differentiation and in adult MuSC following an environmental stimulus to induce stem cell activation and muscle regeneration. Furthermore, the regulation of methylation and demethylation may be dependent on cellular metabolism since availability of the methyl group (CH_3_) is derived from S-adenosyl methionine (SAM), which is converted to S-adenosyl homocysteine (SAH), while Tet dependent demethylation relies heavily on the tricarboxylic acid (TCA) cycle intermediate *α*-ketoglutarate (*α*KG), which is converted to succinate.

The processes of DNA methylation and demethylation are carefully regulated by a family of DNA methyltransferases (DNMTs) and demethylases (the ten-eleven translocation (TET) enzymes) (Figure 1). The methyltransferases DNMT3a and DNMT3b are primarily responsible for the generation of* de novo* DNA methylation [12], while DNMT1 has been found to maintain the methylation patterns following mitosis [13]. Interestingly, while the vast majority of DNA methylation is limited to CpG pairs, several recent studies have identified a significant proportion of CpH (H = A/C/T) methylation sites in a range of cells and tissues, including skeletal muscle and neurons [14, 15]. In neurons, CpH methylation was found to be DNMT3a dependent and was observed to lead to gene repression [15]. In contrast to DNMT enzymes, the TET1, TET2, and TET3 isoforms convert the 5mC to 5-hydroxymethyl cytosine (5hmC, as well as 5-formylcytosine (5fC) and 5-carboxylcytosine (5caC)), which can then be removed through base excision repair mechanisms [16, 17].

DNA methylation was originally thought to occur exclusively during germ cell development and in preimplantation embryos [21, 22]. It is now clear that methylation events occur in response to a variety of environmental cues and may play a larger role in regulating adaptation in adult tissues throughout the lifespan [21, 22]. An overall increase in DNA methylation has been observed as embryonic stem cells transition to late stage progenitor cells and fully differentiated somatic cells [23–25]. These methylation events likely mediate the silencing of gamete specific and pluripotency genes in the transition towards a specific cellular identity. While less common, the loss of methylation can occur in a loci-specific manner to further drive specification [26–28]. These observations provide evidence for a role of transient epigenetic patterning in cell fate decisions and lineage pathways. Whether these epigenetic patterns can be manipulated or even reversed to withdraw differentiated cells from commitment and back towards pluripotency will be of significant focus in the epigenetic and stem cell fields.

## 3. Transcriptional Regulation of Skeletal Muscle Stem Cells

Skeletal muscle is derived from a population of mesodermal progenitor cells that undergo proliferation, differentiation, fusion, and maturation to form skeletal muscle fibers, a process known as myogenesis. Importantly, a subpopulation of these cells exit the cell cycle early and enter a state of quiescence (*G*_0_). These cells are located between the basal lamina and sarcolemma of adult muscle fibers and make up the adult muscle stem cell (MuSC) population (also known as satellite cells). It is this population of cells that confer the high regenerative capacity characteristic of adult skeletal muscle, which in response to injury or trauma become activated and enter the myogenic program to generate new muscle fibers.

The paired domain homeobox 3 (Pax3) transcription factor is critical for successful migration of myogenic progenitor cells to the developing limb bud and subsequent muscle formation [29], while the closely related Pax7 is absolutely critical for the maintenance of the adult MuSC population [30–32]. In addition to Pax3/7, developmental myogenesis is primarily controlled through the actions of the myogenic regulatory factor (MRF) family of transcription factors. The MRFs are basic helix-loop-helix (bHLH) proteins and include myogenic factor 5 (Myf5), myogenic differentiation 1 (MyoD), myogenin, and myogenic regulatory factor 4 (MRF4). The MRFs undergo a strict program of spatial and temporal expression during development to control an array of muscle-specific genes to drive cell identity. The earliest detection of MRF proteins occurs during mid-to-late gestation and is characterized by the appearance of Myf5, closely followed by MyoD [29, 33–35]. These two proteins drive proliferation of the myogenic progenitors and initiate myogenic specification. Myogenin expression soon follows, leading to cells exiting the cell cycle and undergoing terminal differentiation [36, 37]. Fusion and maturation of these cells are regulated (at least in part) by MRF4, which plays a major role in primary and secondary fiber formation [29, 33, 38–40]. From this brief description of the transcriptional regulation of MuSCs, it is clear that the activation of specific transcriptional pathways must be carefully regulated, both spatially and temporally, as cells shift from proliferation to differentiation to a mature muscle fiber.

Indeed, in skeletal muscle biology, one of the most intriguing and pressing questions relates to the processes of MuSC activation, specification to the myogenic lineage, and eventual differentiation. Several studies have provided important evidence linking methylation of the promoter and enhancer regions of myogenic regulators to the initiation of the myogenic transcriptional program in the somites [41, 42]. Although whole genome methylation patterns have been reported in adult skeletal muscle, this type of comprehensive analysis has not yet been applied to the early stages of skeletal muscle development or in purified populations of adult MuSCs. The following section will detail the research to date regarding DNA methylation and its role in mature skeletal muscle function as well as MuSC specification, proliferation, and differentiation.

## 4. Methylation and Skeletal Muscle Stem Cells

### 4.1. DNA Methylation and Demethylation in Quiescent, Proliferating, and Differentiating MuSCs

The differential regulation of DNMT and TET expression and activity during muscle development is critical for understanding the link between environmental cues, intracellular signaling, DNA methylation, and gene expression. Evidence suggests that these methyltransferases and demethylases may be regulated in an isoform-specific manner during myogenesis. Indeed,* Dnmt1* has been found to be downregulated during myogenic differentiation with alternative isoforms of* Dnmt1* and* Dnmt3b* detected specifically in mature skeletal muscle [43–45]. In addition, RNA microarray data has shown that* Tet1* and* Tet2* have increased expression in myoblasts and myotubes in culture when compared with 19 other cell types [46]. In support of elevated demethylase activity in muscle maturation, these authors also reported increased 5hmC levels in adult muscle compared with myoblasts or myotubes [47]. Interestingly, a recently published whole transcriptome dataset from quiescent and proliferating MuSCs showed the nonspecific downregulation of all* Tet* isoforms as well as* Dnmt3a* during MuSC activation, while the expression of* Dnmt1* was robustly increased [18]. These observations suggest that specific DNMT and TET isoforms may be critical for initiating the MRF transcriptional program and/or regulating cell cycle in the transition between quiescence and proliferation and from proliferation to differentiation.

Advances in fluorescent activated cell sorting (FACS) techniques, coupled with downstream gene arrays (Affymetrix microarrays) or whole transcriptome sequencing (RNAseq), have allowed for the generation of transcriptome signatures for pure stem cell populations, including MuSCs [18–20]. A careful analysis of the extensive datasets from these studies reveals a clear pattern of expression for* Dnmt* and* Tet* genes (Table 1). In one such dataset from Ryall and colleagues, the expression of* Dnmt1* was found to increase fourfold and* Dnmt3a* decreased threefold in MuSCs activated* ex vivo*. A similar change in* Dnmt1* and* Dnmt3a* gene expression was observed in two other studies using both* in vivo* and* ex vivo* activated MuSCs [19, 20]. In contrast,* Dnmt3b* expression was unchanged in response to MuSC activation. Interestingly, MuSC activation has been associated with a 2–10-fold decrease in the expression of* Tet1*–*3* genes (Table 1). Together, these results support the need for a direct measurement of the methylation status in quiescent versus actively proliferating MuSCs.

While the methylation status of quiescent MuSCs has not been investigated in detail, several authors have attempted to define a DNA methylation signature in proliferating versus differentiating MuSC cultures. Tsumagari et al. (2013) assessed DNA methylation in proliferating human myoblasts and differentiated myotubes but did not find significant differences between methylation patterns [47]. However, when the DNA methylation profiles of proliferating and differentiating myogenic cells were compared with adult skeletal muscle, they reported a loss of ~90% of the hypermethylated sites in mature fibers [47] with similar findings reported by Carrió et al. (2015) [48]. Interestingly, many of the demethylated genes were associated with homeobox and Tbox transcription factors. Tsumagari and colleagues also reported hypermethylation of the* Pax3* gene in both myogenic cells and mature skeletal muscle. Given the role of Pax3 in migration and early lineage commitment, it would perhaps be interesting, and more informative, to determine the methylation status of this gene during somitogenesis and early specification [47]. Two additional genes observed to be differentially methylated were* Obscn* (encoding a giant muscle associated protein) and* Myh7b* (the gene encoding the slow, cardiac myosin heavy chain) which were both demethylated [47]. In contrast, Miyata et al. (2015) found a small but significant increase in global DNA methylation as myogenesis progressed from myoblast to myotube stage. Gene ontology analysis showed hypermethylation of promoter regions was associated with genes involved in muscle contraction and other muscle processes. Furthermore, two binding motifs recognized by the transcription factors ID4 and ZNF238 were significantly enriched in hypermethylated promoter regions [49]. An important consideration, however, is that the methods used in this study did not distinguish between 5mC and 5hmC. This distinction will be critical for future studies when interpreting the functional impact of methylation changes and the role of 5hmC in gene regulation.

The specific enrichment of 5hmC in either gene bodies or enhancer regions is often associated with activation and has been identified in human embryonic stem cells [50]. In a recent study from Terragni and colleagues, the presence of 5mC and 5hmC within specific gene regions of the Notch signaling pathway was assessed in myoblasts, myotubes, and mature skeletal muscle [51]. Paracrine Notch signaling is critical for the regulation of several developmental pathways, including the proliferation of MuSCs [52]. Using genome-wide profiles of DNA methylation, Terragni et al. identified hypomethylated regions within or near Notch signaling genes including* Notch1* and its ligands* Dll1* and* Jag2* in all skeletal muscle lineages compared with other cell types [51]. Subsequent enzymatic assays revealed enrichment of 5hmC in or near these same genes in mature skeletal muscle, but not myoblasts or myotubes [51]. The 5hmC modification in this context may function as a fine tuning mechanism for rapid induction of gene expression and intercellular signaling to the MuSC niche when skeletal muscle regeneration and/or repair is required.

Brunk and colleagues were the first to perform studies that linked DNA methylation to muscle cell identity [41]. In this study, it was shown that the distal enhancer of* Myod1*, located 20 Kb upstream of the transcriptional start site, was completely unmethylated at all CpG sites examined in myogenic cells and a subpopulation of somite cells. Furthermore, nonmyogenic cells displayed methylation of the enhancer at an average level of >50% [41]. Importantly, the lack of methylation was found to be sufficient for activation of the gene during embryogenesis. More recently, Palacios et al. reported that demethylation of the myogenin promoter occurs in an anterior-posterior manner in cells during somitogenesis, which correlated with myogenin expression and subsequent muscle development [42]. Lucarelli et al. also reported that the myogenin promoter is unmethylated in differentiated muscle cells and correlates with its expression [53].

Since the seminal work by Brunk and colleagues in 1996, Carrió et al. investigated the methylation status of a 110 kb enhancer region of Myf5/Myf6 (known as a “super-enhancer” because it has a high density of enhancer elements) [54]. Of the five enhancer elements analyzed within this region, all were highly methylated in ESCs and almost totally demethylated in myoblasts, myotubes, and skeletal muscle in concert with increased Myf5 gene expression [48]. Importantly, these were muscle-specific observations and localized to the enhancer regions [48]. Together these findings suggest that DNA methylation/demethylation plays a critical role in regulating gene expression to control muscle cell specification and highlights an important role in gene regulation for DNA methylation changes outside promoter regions.

To better characterize the role of DNA demethylation in myogenic development, several studies have utilized 5-azacytidine (5AC), a potent inhibitor of DNA methylation, which acts via the sequestration of DNMT1 and results in global loss of methylation. Mouse fibroblasts (C3H10T1/2) treated with 5AC for 10 days resulted in the emergence of several cell types including those of adipogenic and osteogenic lineages. However, the majority of cells underwent transformation towards the myogenic lineage [55]. Similar results have been observed in fibroblasts following DNMT1 inhibition via antisense RNA [56]. Together, these findings provide strong evidence that DNA methylation plays an important role in dictating cell fate.

In the immortalized C2C12 myogenic cell line, proliferating myoblasts treated with 5AC exhibited increased expression of muscle-specific genes (including myogenin), enhanced myotube maturation, spontaneous contraction, and Ca^2+^ transients [57–59]. These results suggest that DNA demethylation in cells already committed to the myogenic lineage likely induces a permissive chromatin configuration, allowing muscle-specific transcription factors to bind their target gene promoters to promote differentiation. In similar experiments with 5AC, increased protein expression of cyclin D (linked to differentiation) and p21 (associated with the maintenance of the postmitotic state), as well as the gene expression of the myogenic regulators* Myf5* and* Myod1*, has been observed [60]. These findings support a role for DNA demethylation during myogenic differentiation. However, as these studies were conducted in proliferating myoblasts that are already committed to the myogenic lineage, it is important that future studies investigate DNA methylation in each broad step of myogenesis (quiescence, proliferation, and differentiation). Furthermore, genome-wide methylation analysis such as reduced representation bisulfite sequencing will provide comprehensive and important information regarding the earliest stages of muscle development and regeneration.

### 4.2. *Dnmt* and* Tet* Enzymes in Embryonic Development

The generation of* Tet* and* Dnmt* isoform-specific knockout (KO) mice has greatly enhanced our understanding of DNA methylation in embryonic stem cells and development. Of particular interest, the deletion of TET1 in embryonic stem cells results in reduced 5hmC and dysregulation of 221 genes, including muscle development and contractile genes [61]. However,* Tet1* KO mice remain viable with only a slightly reduced body size. Similarly, loss of* Tet2* also results in viable mice; however, these animals display myeloid malignancies associated with the dysregulation of hematopoietic stem cells as a result of the loss of 5hmC and elevated levels of 5mC in bone marrow cells [62, 63]. In embryonic stem cells lacking both* Tet1* and* Tet2*, there is a greater loss of 5hmC than that observed in cells lacking only* Tet1* or* Tet2*, but these cells still remain pluripotent. The resulting double KO mice demonstrate partial perinatal lethality, with those surviving mice displaying reduced fertility [64]. Finally, loss of* Tet3* leads to neonatal lethality with abnormal hydroxylation and impaired demethylation of the paternal genome [65]. These findings suggest that the TET isoforms functions are not redundant and play specific roles in cell fate decisions and organ development. In contrast to the loss of either* Tet1* or* Tet2*, DNMTs appear to be far more critical for survival.* Dnmt1* KO embryos arrest at the 8th somite stage and display ~70% reduction in methylation levels [66] and* Dnmt3a* KO mice survive only to ~4 weeks of age, while* Dnmt3b* KO mice are not viable [67]. Altogether, it is clear that regulation of DNA methylation is critical for embryonic development and DNMT and TET enzymes play important and potentially tissue specific roles in cell fate. Future studies taking advantage of conditional and inducible KO models will be crucial to dissect the complex interaction of DNA methylation and demethylation in the regulation of transcriptional networks and tissue development.

### 4.3. A Novel Role for Metabolism in the Regulation of MuSC DNA Methylation

In addition to the differential regulation of* Dnmt* and* Tet* expression during myogenesis, the activity of these proteins can be regulated in a metabolic dependent manner (Figure 1) [68, 69]. The process of DNA methylation involves the attachment of a methyl group (-CH_3_) to the 5′ position of a cytosine base. This methyl group is derived from S-adenosyl methionine (SAM), which is in turn produced via one-carbon metabolism (specifically the folate and methionine cycles). The precursors necessary to produce SAM for DNA methylation are derived from the diet (folate) or glycolysis (3-phosphoglycerate (3PG)→serine→glycine + 5,10-methylenetetrahydrofolate (metTHF)). In contrast, TET dependent DNA demethylation requires the tricarboxylic acid (TCA) cycle intermediate *α*-ketoglutarate (*α*KG) to proceed. This dependency of both DNMT and TET proteins on metabolites suggests that significant changes in cellular metabolism may be associated with dramatic changes in the cellular DNA methylation patterns [70].

Recent work has identified a process of metabolic reprogramming in MuSCs as they move from quiescence to proliferation, with fatty-acid oxidation predominating during quiescence and glycolysis increasing during proliferation [18]. While this process of metabolic reprogramming was linked to altered transcription and gene expression as a result of increased histone acetylation, it seems likely that such a dramatic shift in metabolism (and cell state) would likely be associated with several epigenetic changes, including DNA methylation. Of particular interest to the current discussion are findings suggesting that the shift from quiescence to proliferation in MuSCs is associated with a significant increase in the expression of genes associated with the enzymatic conversion of 3PG to metTHF (phosphoglycerate dehydrogenase,* Phgdh*; phosphoserine aminotransferase 1,* Psat1*; phosphoserine phosphatase,* Psph*; and serine hydroxymethyltransferase,* Shmt*) and a decrease in the expression of isocitrate dehydrogenase 1 (*Idh1*, responsible for the conversion of isocitrate to *α*KG). These changes in the expression level of key metabolic enzymes, coupled with the previously identified changes in* Dnmt* and* Tet* (Table 1), suggest a likely increase in DNA methylation during MuSC activation/proliferation [18, 20]. Future studies investigating the link between the metabolic switch during MuSC activation and changes in DNA methylation will be fundamental for our understanding of MuSC specification and the downstream transcriptional program.

## 5. Skeletal Muscle DNA Methylation and Physical Activity

Several studies have compared CpG methylation patterns in adult skeletal muscle to that in other tissue types in order to define the DNA methylation signature of skeletal muscle. One human study assessed seventeen thousand CpG islands of which 178 were specifically hypermethylated in skeletal muscle compared with other cell types including blood, sperm, brain, and spleen [71]. A similar study identified 183 differentially methylated CpG sites in 22 skeletal muscle samples, within a set of 1,628 human tissues [72]. Finally, Calvanese et al. identified 47 genes that were hypomethylated exclusively in skeletal muscle, with some of these encoding contractile proteins such as obscurins, myotilin, and the slow-twitch myosin heavy chain [27]. Together, these findings have clearly demonstrated that different tissue types display distinct DNA methylation patterns appropriate for genetic control of their function and structure, but they fail to provide information regarding the dynamic methylation processes that may occur in response to environmental stimuli or during tissue development.

It is now accepted that DNA methylation is a dynamic process, and as skeletal muscle is a highly plastic tissue able to rapidly respond to changes in demand, DNA methylation may be a particularly important mediator of these adaptations. Skeletal muscle responds to endurance and resistance training through adaptation of contractile apparatus and metabolic capacity. Barrès and colleagues have previously reported that acute exercise, in humans and mice, is linked to transient DNA demethylation in the promoter region of genes including peroxisome proliferator activated receptor *γ* (*Pparg*) coactivator-1*α* (*Ppargc1a*), pyruvate dehydrogenase kinase 4 (*Pdk4*), and* Ppard* in skeletal muscle, which corresponded to transient induction of gene expression in a time and intensity dependent manner [73]. This data suggests that at least part of skeletal muscle adaptation to exercise/contraction may be mediated through transient regulation of DNA methylation. Others have provided evidence that histone modifications also occur in human skeletal muscle following an acute bout of exercise [74], while long-term training may cause sustained impacts on DNA methylation patterns of muscle-specific genes [75].

There is also mounting evidence that during perinatal development skeletal muscle is susceptible to insults or stimuli that may alter the epigenetic program, which has consequences for gene transcription and functional outcomes later in life [76–80]. For example, in mice, obesity in the mother caused DNA hypermethylation at the promoter of the metabolic master regulator,* Ppargc1a*, in skeletal muscle of the offspring [76]. This was detected at birth and up to 12 months of age and led to functional consequences for* Ppargc1a* mRNA levels and downstream gene expression (*Glut4*,* Cox4*, and* CytC*) [76]. Furthermore, these epigenetic changes were associated with metabolic dysfunction later in life [76]. Interestingly, when the obese mother was allowed to exercise prior to and during pregnancy, the hypermethylation of the promoter region of* Ppargc1a* was abolished in the skeletal muscle of the offspring along with the associated functional consequences [76]. It is therefore possible, and highly likely, that other genes important for skeletal muscle development may be epigenetically regulated in early life and susceptible to environmental stimuli during critical periods of cell growth.

## 6. Conclusions

Additional studies are warranted to further characterize how DNA methylation and hydroxymethylation differ between MuSCs in different dynamic states and what specifically regulates these methylation events. In doing so, these studies will reveal novel mechanisms to regulate MuSC identity and growth. Furthermore, the identification of isoform-specific roles for* Dnmt* and* Tet* enzymes in regulating the MRF transcriptional program would provide new insight into DNA methylation dynamics and MuSC function and could be extended to investigate periods of muscle adaptation and plasticity. Finally, whole genome sequencing will allow us to take this research beyond the classical muscle-specific genes and also extend our reach to regions of intra- and intergenic DNA methylation in regulating transcriptional programs. These findings will be crucial for furthering our fundamental understanding of stem cell biology and epigenetic regulation and may lead to the development of novel techniques to induce pluripotency in committed cells and unveil new therapeutic targets.

## Figures and Tables

**Table 1:** A summary of differential gene expression in DNA methyltransferases and demethylases following MuSC activation (fold change compared to quiescent MuSCs).

	Ryall et al. 2015 [18] (RNAseq, *in vitro* MuSC activation)	Pallafacchina et al. 2010 [19] (microarray, *in vitro* MuSC activation)	Liu et al. 2013 [20] (microarray, *in vivo *MuSC activation)	Pallafacchina et al. 2010 [19] (microarray, MuSCs from one-week-old mice)	Pallafacchina et al. 2010 [19] (microarray, MuSCs from *mdx* dystrophic mice)
*Dnmt1*	↑ 4-fold	↑ 6-fold	↑ 6-fold	↑ 7-fold	↑ 3-fold
*Dnmt3a*	↓ 3-fold	↓ 3-fold	NA	↑ 3-fold	*↔*
*Dnmt3b*	*↔*	NA	NA	NA	NA
*Tet1*	↓ 10-fold	NA	↓ 5-fold	NA	NA
*Tet2*	↓ 2-fold	↓ 13-fold	↓ 3-fold	NA	NA
*Tet3*	↓ 2-fold	NA	NA	NA	NA

DNMT: DNA methyltransferase; Tet: ten-eleven translocase; MuSC: muscle stem cell; NA: not available.

## References

[1] Hochedlinger K., Plath K. (2009). Epigenetic reprogramming and induced pluripotency. *Development*.

[2] Bannister A. J., Kouzarides T. (2011). Regulation of chromatin by histone modifications. *Cell Research*.

[3] Dixon J. R., Jung I., Selvaraj S. (2015). Chromatin architecture reorganization during stem cell differentiation. *Nature*.

[4] Kundaje A., Meuleman W., Ernst J. (2015). Integrative analysis of 111 reference human epigenomes. *Nature*.

[5] Smith Z. D., Chan M. M., Humm K. C. (2014). DNA methylation dynamics of the human preimplantation embryo. *Nature*.

[6] Bogdanović O., Veenstra G. J. C. (2009). DNA methylation and methyl-CpG binding proteins: developmental requirements and function. *Chromosoma*.

[7] Maunakea A. K., Chepelev I., Cui K., Zhao K. (2013). Intragenic DNA methylation modulates alternative splicing by recruiting MeCP2 to promote exon recognition. *Cell Research*.

[8] Sati S., Tanwar V. S., Kumar K. A. (2012). High resolution methylome map of rat indicates role of intragenic DNA methylation in identification of coding region. *PLoS ONE*.

[9] Shukla S., Kavak E., Gregory M. (2011). CTCF-promoted RNA polymerase II pausing links DNA methylation to splicing. *Nature*.

[10] Weber M., Hellmann I., Stadler M. B. (2007). Distribution, silencing potential and evolutionary impact of promoter DNA methylation in the human genome. *Nature Genetics*.

[11] Schmidl C., Klug M., Boeld T. J. (2009). Lineage-specific DNA methylation in T cells correlates with histone methylation and enhancer activity. *Genome Research*.

[12] Bird A. (2002). DNA methylation patterns and epigenetic memory. *Genes and Development*.

[13] Trasler J., Deng L., Melnyk S. (2003). Impact of Dnmt1 deficiency, with and without low folate diets, on tumor numbers and DNA methylation in min mice. *Carcinogenesis*.

[14] Barrès R., Osler M. E., Yan J. (2009). Non-CpG methylation of the PGC-1alpha promoter through DNMT3B controls mitochondrial density. *Cell Metabolism*.

[15] Guo J. U., Su Y., Shin J. H. (2014). Distribution, recognition and regulation of non-CpG methylation in the adult mammalian brain. *Nature Neuroscience*.

[16] Tahiliani M., Koh K. P., Shen Y. (2009). Conversion of 5-methylcytosine to 5-hydroxymethylcytosine in mammalian DNA by MLL partner TET1. *Science*.

[17] Ito S., D'Alessio A. C., Taranova O. V., Hong K., Sowers L. C., Zhang Y. (2010). Role of Tet proteins in 5mC to 5hmC conversion, ES-cell self-renewal and inner cell mass specification. *Nature*.

[18] Ryall J. G., Dell'Orso S., Derfoul A. (2015). The NAD^+^-dependent SIRT1 deacetylase translates a metabolic switch into regulatory epigenetics in skeletal muscle stem cells. *Cell Stem Cell*.

[19] Pallafacchina G., François S., Regnault B. (2010). An adult tissue-specific stem cell in its niche: a gene profiling analysis of in vivo quiescent and activated muscle satellite cells. *Stem Cell Research*.

[20] Liu L., Cheung T. H., Charville G. W. (2013). Chromatin modifications as determinants of muscle stem cell quiescence and chronological aging. *Cell Reports*.

[21] Reik W. (2007). Stability and flexibility of epigenetic gene regulation in mammalian development. *Nature*.

[22] Bhutani N., Burns D. M., Blau H. M. (2011). DNA demethylation dynamics. *Cell*.

[23] Fouse S. D., Shen Y., Pellegrini M. (2008). Promoter CpG methylation contributes to ES cell gene regulation in parallel with Oct4/Nanog, PcG complex, and histone H3 K4/K27 trimethylation. *Cell Stem Cell*.

[24] Meissner A., Mikkelsen T. S., Gu H. (2008). Genome-scale DNA methylation maps of pluripotent and differentiated cells. *Nature*.

[25] Isagawa T., Nagae G., Shiraki N. (2011). DNA methylation profiling of embryonic stem cell differentiation into the three germ layers. *PLoS ONE*.

[26] Nagae G., Isagawa T., Shiraki N. (2011). Tissue-specific demethylation in CpG-poor promoters during cellular differentiation. *Human Molecular Genetics*.

[27] Calvanese V., Fernández A. F., Urdinguio R. G. (2012). A promoter DNA demethylation landscape of human hematopoietic differentiation. *Nucleic Acids Research*.

[28] Nazor K. L., Altun G., Lynch C. (2012). Recurrent variations in DNA methylation in human pluripotent stem cells and their differentiated derivatives. *Cell Stem Cell*.

[29] Tajbakhsh S., Rocancourt D., Cossu G., Buckingham M. (1997). Redefining the genetic hierarchies controlling skeletal myogenesis: *Pax-3* and *Myf-5* act upstream of *MyoD*. *Cell*.

[30] Seale P., Sabourin L. A., Girgis-Gabardo A., Mansouri A., Gruss P., Rudnicki M. A. (2000). Pax7 is required for the specification of myogenic satellite cells. *Cell*.

[31] Oustanina S., Hause G., Braun T. (2004). Pax7 directs postnatal renewal and propagation of myogenic satellite cells but not their specification. *The EMBO Journal*.

[32] Relaix F., Rocancourt D., Mansouri A., Buckingham M. (2005). A Pax3/Pax7-dependent population of skeletal muscle progenitor cells. *Nature*.

[33] Braun T., Arnold H.-H. (1995). Inactivation of Myf-6 and Myf-5 genes in mice leads to alterations in skeletal muscle development. *The EMBO Journal*.

[34] Gerhart J., Elder J., Neely C. (2006). MyoD-positive epiblast cells regulate skeletal muscle differentiation in the embryo. *Journal of Cell Biology*.

[35] Cossu G., Kelly R., Tajbakhsh S., Di Donna S., Vivarelli E., Buckingham M. (1996). Activation of different myogenic pathways: myf-5 is induced by the neural tube and MyoD by the dorsal ectoderm in mouse paraxial mesoderm. *Development*.

[36] Rawls A., Morris J. H., Rudnicki M. (1995). Myogenin's functions do not overlap with those of MyoD or Myf-5 during mouse embryogenesis. *Developmental Biology*.

[37] Hasty P., Bradley A., Morris J. H. (1993). Muscle deficiency and neonatal death in mice with a targeted mutation in the myogenin gene. *Nature*.

[38] Thompson W. J., Condon K., Astrow S. H. (1990). The origin and selective innervation of early muscle fiber types in the rat. *Journal of Neurobiology*.

[39] Zhang M., McLennan I. S. (1995). During secondary myotube formation, primary myotubes preferentially absorb new nuclei at their ends. *Developmental Dynamics*.

[40] Kassar-Duchossoy L., Gayraud-Morel B., Gomès D. (2004). Mrf4 determines skeletal muscle identity in Myf5:Myod double-mutant mice. *Nature*.

[41] Brunk B. P., Goldhamer D. J., Emerson C. P. (1996). Regulated demethylation of the myoD distal enhancer during skeletal myogenesis. *Developmental Biology*.

[42] Palacios D., Summerbell D., Rigby P. W. J., Boyes J. (2010). Interplay between DNA methylation and transcription factor availability: implications for developmental activation of the mouse *Myogenin* gene. *Molecular and Cellular Biology*.

[43] Liu Y., Sun L., Jost J.-P. (1996). In differentiating mouse myoblasts DNA methyltransferase is posttranscriptionally and posttranslationally regulated. *Nucleic Acids Research*.

[44] Aguirre-Arteta A. M., Grunewald I., Cardoso M. C., Leonhardt H. (2000). Expression of an alternative Dnmt1 isoform during muscle differentiation. *Cell Growth and Differentiation*.

[45] Robertson K. D., Uzvolgyi E., Liang G. (1999). The human DNA methyltransferases (DNMTs) 1, 3a and 3b: coordinate mRNA expression in normal tissues and overexpression in tumors. *Nucleic Acids Research*.

[46] Tsumagari K., Chang S.-C., Lacey M. (2011). Gene expression during normal and FSHD myogenesis. *BMC Medical Genomics*.

[47] Tsumagari K., Baribault C., Terragni J. (2013). Early de novo DNA methylation and prolonged demethylation in the muscle lineage. *Epigenetics*.

[48] Carrió E., Díez-Villanueva A., Lois S. (2015). Deconstruction of DNA methylation patterns during myogenesis reveals specific epigenetic events in the establishment of the skeletal muscle lineage. *Stem Cells*.

[49] Miyata K., Miyata T., Nakabayashi K. (2015). DNA methylation analysis of human myoblasts during in vitro myogenic differentiation: de novo methylation of promoters of muscle-related genes and its involvement in transcriptional down-regulation. *Human Molecular Genetics*.

[50] Stroud H., Feng S., Morey Kinney S., Pradhan S., Jacobsen S. E. (2011). 5-Hydroxymethylcytosine is associated with enhancers and gene bodies in human embryonic stem cells. *Genome Biology*.

[51] Terragni J., Zhang G., Sun Z. (2014). Notch signaling genes: myogenic DNA hypomethylation and 5-hydroxymethylcytosine. *Epigenetics*.

[52] Qin L., Xu J., Wu Z. (2013). Notch1-mediated signaling regulates proliferation of porcine satellite cells (PSCs). *Cellular Signalling*.

[53] Lucarelli M., Fuso A., Strom R., Scarpa S. (2001). The dynamics of myogenin site-specific demethylation is strongly correlated with its expression and with muscle differentiation. *The Journal of Biological Chemistry*.

[54] Whyte W. A., Orlando D. A., Hnisz D. (2013). Master transcription factors and mediator establish super-enhancers at key cell identity genes. *Cell*.

[55] Constantinides P. G., Jones P. A., Gevers W. (1977). Functional striated muscle cells from non myoblast precursors following 5-azacytidine treatment. *Nature*.

[56] Szyf M., Rouleau J., Theberge J., Bozovic V. (1992). Induction of myogenic differentiation by an expression vector encoding the DNA methyltransferase cDNA sequence in the antisense orientation. *The Journal of Biological Chemistry*.

[57] Hupkes M., van Someren E. P., Middelkamp S. H. A., Piek E., van Zoelen E. J., Dechering K. J. (2011). DNA methylation restricts spontaneous multi-lineage differentiation of mesenchymal progenitor cells, but is stable during growth factor-induced terminal differentiation. *Biochimica et Biophysica Acta: Molecular Cell Research*.

[58] Hupkes M., Jonsson M. K. B., Scheenen W. J. (2011). Epigenetics: DNA demethylation promotes skeletal myotube maturation. *The FASEB Journal*.

[59] Scarpa S., Lucarelli M., Palitti F., Carotti D., Strom R. (1996). Simultaneous myogenin expression and overall DNA hypomethylation promote in vitro myoblast differentiation. *Cell Growth and Differentiation*.

[60] Montesano A., Luzi L., Senesi P., Terruzzi I. (2013). Modulation of cell cycle progression by 5-azacytidine is associated with early myogenesis induction in murine myoblasts. *International Journal of Biological Sciences*.

[61] Dawlaty M. M., Ganz K., Powell B. E. (2011). Tet1 is dispensable for maintaining pluripotency and its loss is compatible with embryonic and postnatal development. *Cell Stem Cell*.

[62] Li Z., Cai X., Cai C.-L. (2011). Deletion of Tet2 in mice leads to dysregulated hematopoietic stem cells and subsequent development of myeloid malignancies. *Blood*.

[63] Moran-Crusio K., Reavie L., Shih A. (2011). *Tet2* loss leads to increased hematopoietic stem cell self-renewal and myeloid transformation. *Cancer Cell*.

[64] Dawlaty M. M., Breiling A., Le T. (2013). Combined deficiency of Tet1 and Tet2 causes epigenetic abnormalities but is compatible with postnatal development. *Developmental Cell*.

[65] Gu T.-P., Guo F., Yang H. (2011). The role of Tet3 DNA dioxygenase in epigenetic reprogramming by oocytes. *Nature*.

[66] Li E., Bestor T. H., Jaenisch R. (1992). Targeted mutation of the DNA methyltransferase gene results in embryonic lethality. *Cell*.

[67] Okano M., Bell D. W., Haber D. A., Li E. (1999). DNA methyltransferases Dnmt3a and Dnmt3b are essential for de novo methylation and mammalian development. *Cell*.

[68] Locasale J. W. (2013). Serine, glycine and one-carbon units: cancer metabolism in full circle. *Nature Reviews Cancer*.

[69] Teperino R., Schoonjans K., Auwerx J. (2010). Histone methyl transferases and demethylases; can they link metabolism and transcription?. *Cell Metabolism*.

[70] Carey B. W., Finley L. W. S., Cross J. R., Allis C. D., Thompson C. B. (2014). Intracellular *α*-ketoglutarate maintains the pluripotency of embryonic stem cells. *Nature*.

[71] Illingworth R., Kerr A., DeSousa D. (2008). A novel CpG island set identifies tissue-specific methylation at developmental gene loci. *PLoS Biology*.

[72] Fernandez A. F., Assenov Y., Martin-Subero J. I. (2012). A DNA methylation fingerprint of 1628 human samples. *Genome Research*.

[73] Barrès R., Yan J., Egan B. (2012). Acute exercise remodels promoter methylation in human skeletal muscle. *Cell Metabolism*.

[74] McGee S. L., Fairlie E., Garnham A. P., Hargreaves M. (2009). Exercise-induced histone modifications in human skeletal muscle. *Journal of Physiology*.

[75] Nitert M. D., Dayeh T., Volkov P. (2012). Impact of an exercise intervention on DNA methylation in skeletal muscle from first-degree relatives of patients with type 2 diabetes. *Diabetes*.

[76] Laker R. C., Lillard T. S., Okutsu M. (2014). Exercise prevents maternal high-fat diet-induced hypermethylation of the *Pgc-1α* gene and age-dependent metabolic dysfunction in the offspring. *Diabetes*.

[77] Zheng S., Rollet M., Pan Y.-X. (2011). Maternal protein restriction during pregnancy induces CCAAT/enhancer-binding protein (C/EBP*β*) expression through the regulation of histone modification at its promoter region in female offspring rat skeletal muscle. *Epigenetics*.

[78] Bharathy N., Ling B. M. T., Taneja R. (2013). Epigenetic regulation of skeletal muscle development and differentiation. *Subcellular Biochemistry*.

[79] Brøns C., Jacobsen S., Nilsson E. (2010). Deoxyribonucleic acid methylation and gene expression of *PPARGC1A* in human muscle is influenced by high-fat overfeeding in a birth-weight-dependent manner. *Journal of Clinical Endocrinology and Metabolism*.

[80] Raychaudhuri N., Raychaudhuri S., Thamotharan M., Devaskar S. U. (2008). Histone code modifications repress glucose transporter 4 expression in the intrauterine growth-restricted offspring. *The Journal of Biological Chemistry*.

